# Discrepancies between clinical diagnosis and hospital autopsy: A comparative retrospective analysis of 1,112 cases

**DOI:** 10.1371/journal.pone.0255490

**Published:** 2021-08-13

**Authors:** Stephan D. Kurz, Viyan Sido, Hermann Herbst, Bernhard Ulm, Erma Salkic, Thomas M. Ruschinski, Claas T. Buschmann, Michael Tsokos

**Affiliations:** 1 German Heart Institute Berlin, Institute for Anaesthesiology, Berlin, Germany; 2 Institute of Physiology, Berlin Institute of Health, Charite–Universitätsmedizin Berlin, Corporate Member of Freie Universität Berlin, Humboldt-Universität zu Berlin, Berlin, Germany; 3 Department of Cardiovascular Surgery, Berlin Institute of Health, Charite–Universitätsmedizin Berlin, Corporate Member of Freie Universität Berlin, Humboldt-Universität zu Berlin, Berlin, Germany; 4 Department of Pathology, Vivantes Klinikum Neukölln, Berlin, Germany; 5 Independent statistical advice, Munich, Germany; 6 Department of Cardiology, Berlin Institute of Health, Charite–Universitätsmedizin Berlin, Corporate Member of Freie Universität Berlin, Humboldt-Universität zu Berlin, Berlin, Germany; 7 Institute of Legal Medicine, University Hospital Schleswig-Holstein Kiel/Lübeck, Lubeck, Germany; 8 Institute of Legal Medicine and Forensic Sciences, Berlin Institute of Health, Charite–Universitätsmedizin Berlin, Corporate Member of Freie Universität Berlin, Humboldt-Universität zu Berlin, Berlin, Germany; Imam Abdulrahman Bin Faisal University, SAUDI ARABIA

## Abstract

**Aims:**

The aim of this study was to compare discrepancies between diagnosed and autopsied causes of death in 1,112 hospital autopsies and to determine the factors causing this discrepancies.

**Methods:**

1,112 hospital autopsies between 2010 and 2013 were retrospectively studied. Ante-mortem diagnoses were compared to causes of death as determined by autopsy. Clinical diagnoses were extracted from the autopsy request form, and post-mortem diagnoses were assessed from respective autopsy reports. Variables, such as sex, age, Body Mass Index, category of disease, duration of hospital stay and new-borns were studied in comparison to discrepancy. P-values were derived from the Mann-Whitney U test for the constant features and chi-2 test, p-values < 0,05 were considered significant.

**Results:**

73.9% (n = 822) patients showed no discrepancy between autopsy and clinical diagnosis. The duration of hospitalisation (6 vs. 9 days) and diseases of the **cardiovascular system** (61.7%) had a significant impact on discrepancies.

**Conclusion:**

Age, **cardiovascular** diseases and duration of hospital stay significantly affect discrepancies in ante- and post-mortem diagnoses.

## Introduction

Autopsies are indispensable in medicine and are important for medical education, quality assurance and to confirm the clinical diagnosis [1–4]. Correct diagnosis is important for patient care, as well as evidence for the number of morbidity and mortality [5]. Hospital autopsies are considered as “gold standard” for providing a definitive diagnosis and usually have been used to compare the clinical diagnosis with post-mortem findings [6]. Despite the significant progress in diagnostic procedures, ante- and postmortem diagnoses differ [7]. Autopsy is not only the last medical service for the patient; in addition to determining causes of death autopsy also helps to clarify the underlying disease of the deceased patient. Antemortem consent of the deceased or postmortem consent of the relatives are mandatory for clinical autopsies (in contrast to forensic autopsies).

The aim of the study was to identify the discrepancies between autopsy findings and clinical diagnosis. Our additional aim was to identify factors that might have had an impact on the occurrence of these discrepancies and whether these discrepancies are higher for some morbidities then others.

## Material and methods

In this retrospective study, reports of all autopsies from 01-01-2010 to 31-12-2013 performed at the Department of Pathology in the Vivantes Hospital Neukölln were reviewed (n = 1,112). The Vivantes Hospital Neukölln is a maximum care hospital and with > 1,200 beds and > 15,000 surgical interventions per year one of the largest hospitals in Berlin, Germany., Clinical diagnoses were extracted from the clinical information written on the autopsy request form, and the post-mortem diagnoses were assessed from the autopsy report. We compared the immediate cause of death, i. e. the main result of the autopsy, and the main clinical diagnosis prior to death for discrepancies, a discrepancy was defined as different ante-/postmortem diagnosis with respect to the cause of death. Furthermore, age and sex, Body Mass Index (BMI), length of in-hospital stay, main and secondary diagnosis and the category of disease were collected.

We examined the relation between the cases with diagnostic discrepancies and the length of hospital stay. Analyses were performed by applying the statistical programme R (Version 3.2.3). Variables, such as sex, age, BMI, category of disease and hospital duration were studied in comparison to discrepancy. In our analyses, the p-values were derived from the Mann-Whitney U test for the continuous features and comparison of proportions between groups was performed with the chi-2 test, P values < 0.05 were considered significant. Categorical variables were described as absolute value and percentage. Multivariate analysis was performed with logistic regression.

### Declaration and ethic approval

The study was approved by the Ethics Committee of the Charité-Universitätsmedizin Berlin (Ethics Subcommittee 2 of Charité-Universitätsmedizin Berlin Campus Virchow-Klinikum, registration number: EA2/126/14) and complies with the Declaration of Helsinki [8].

## Results

The baseline characteristics of the total cohort in Table 1 shows discrepant findings for 290 out of 1112 hospital autopsies. 822 out of 1,112 hospital autopsies showed no discrepancies. Patients with discrepancies were significantly older (aged > 71). Main causes of death at autopsy were diseases of the cardiovascular system especially in discrepant findings (62%). In further categories of diseases, differences are negligible. The analyzed group of new-borns did not reach statistical significance, 108 out of 113 new-borns (first 28 days of life) showed no discrepancies and only five cases were discrepant (P < 0,001).

**Table 1:** Baseline characteristics in the total cohort, illustrating medians (25% quantiles; 75% quantiles), absolute frequencies (%) and p-values originating from Mann-Whitney U tests for continuous variables, Chi-square tests and the clinical characteristics of the group of patients.

	[ALL]	No discrepancy”	Discrepancy	p-value	N*
	N = 1112	N = 822	N = 290		
Sex				0.124	1109
male	644 (58.1%)	464 (56.7%)	180 (62.1%)		
female	465 (41.9%)	355 (43.3%)	110 (37.9%)		
Age	69.0 [55.8;77.0]	68.0 [53.0;77.0]	71.0 [62.0;78.0]	<0.001	1112
BMI	25.7 [21.2;31.1]	25.6 [20.7;31.2]	25.8 [22.5;31.1]	0.060	1112
Category of disease				<0.001	1108
*Cardiovascular system*	421 (38.0%)	242 (29.6%)	179 (61.7%)		
*Tumour*	157 (14.2%)	131 (16.0%)	26 (8.97%)		
*Infectious disease*	162 (14.6%)	145 (17.7%)	17 (5.86%)		
*Respiratory system*	152 (13.7%)	118 (14.4%)	34 (11.7%)		
*Digestive system*	104 (9.39%)	76 (9.29%)	28 (9.66%)		
*Genitourinary system*	6 (0.54%)	6 (0.73%)	0 (0.00%)		
*Other diseases*	106 (9.57%)	100 (12.2%)	6 (2.07%)		
Duration of hospital stay in days (d)	6.00 [1.00;16.0]	7.00 [1.00;17.0]	6.00 [2.00;15.0]	0.903	954
New-borns:				<0.001	1112
yes	113 (10.2%)	108 (13.1%)	5 (1.72%)		
no	999 (89.8%)	714 (86.9%)	285 (98.3%)		

**Fig 1:**
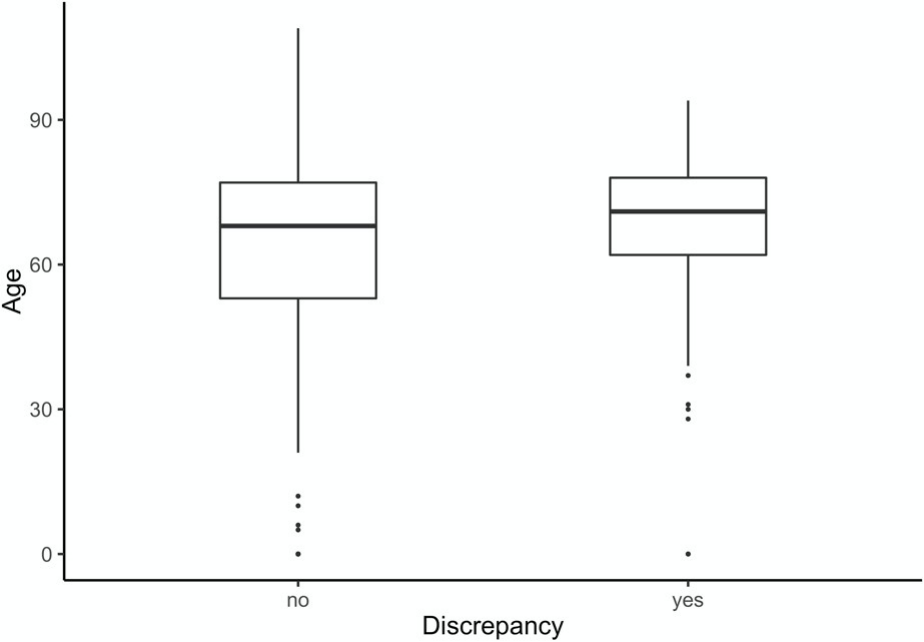
The Box-plot above compares age to discrepancy groups.

Fig 1 demonstrates that the distribution of age from approximately 55–70 years was not discrepant, whereas the age group from 62–70 years shows discrepancies. With increasing age, the diagnoses became more complex and the discrepancies in the final diagnosis were more frequent, especially with shorter duration of hospital stay. To better represent this group, new-borns were excluded.

Table 2 represents the same data (n = 999, new-borns were excluded). Again, patients with cardiovascular diseases also showed on average more discrepancies in the post-mortem hospital autopsy when compared to the clinical diagnosis. The median length of in hospital stay was significantly longer (plus three days) in the group “discrepancy”.

**Table 2:** Groups of discrepancy without the group of new-borns.

	[ALL]	No discrepancy”	Discrepancy	p-value	N*
	N = 999	N = 714	N = 285		
Sex				0.172	999
male	585 (58.6%)	408 (57.1%)	177 (62.1%)		
female	414 (41.4%)	306 (42.9%)	108 (37.9%)		
Age	71.0 [61.0;78.0]	70.0 [60.0;78.0]	71.0 [63.0;78.0]	0.195	999
BMI	26.9 [22.9;31.9]	27.1 [22.9;32.4]	26.1 [22.6;31.1]	0.323	999
Category of disease				<0.001	996
*Cardiovascular system*	417 (41.9%)	239 (33.6%)	178 (62.5%)		
*Tumour*	157 (15.8%)	131 (18.4%)	26 (9.12%)		
*Infectious diseases*	153 (15.4%)	138 (19.4%)	15 (5.26%)		
*Respiratory system*	145 (14.6%)	111 (15.6%)	34 (11.9%)		
*Digestive system*	102 (10.2%)	74 (10.4%)	28 (9.82%)		
*Genitourinary system*	6 (0.60%)	6 (0.84%)	0 (0.00%)		
*Other diseases*	16 (1.61%)	12 (1.69%)	4 (1.40%)		
Duration of hospital stay in days (d)	8.00 [2.00;17.0]	9.00 [2.00;18.0]	6.00 [2.00;15.0]	0.028	869

Table 3 represents the Differences between groups of discrepancy, category of disease and duration of hospitalization of patients. There are no significant differences between the groups. For this purpose, the Mann-Whitney U test was applied. The p-values in columns show the Kruskal Wallis test results of hospital stays in various categories. If the duration of hospital stay is compared within the non- discrepancy group with the category of diseases, a significant difference is present. However, results of the Kruskal Wallis test for all patients and those demonstrating ‘no discrepancy’ were statistically significant.

**Table 3:** Table 3 represents the differences between groups of discrepancy, category of disease and duration of hospitalization of patients.

	Category of Disease	N	All	No Discrepancy	Discrepancy	p-value
1	Cardiovascular system	436	5[2;13]	5[1;12.75]	5[1;14]	0.667
2	Tumour	158	10[4;19]	10[4.20]	9[4;15.75]	0.502
3	Infectious diseases	154	10[4;22.5]	10[4;23]	10[4;17]	0.718
4	Respiratory system	145	9[2.75;21.25]	11[2;21]	7[3.50;21]	0.687
5	Digestive system	107	10[3;22]	11[4;23]	10[1;15]	0.239
6	Other diseases	16	11[5.25;28]	14[3;26]	8[7;22]	0.755
7	Genitourinary system	6	2[1;19]	2[1;19]		
	p-value		<0,0001	<0,0001	0.41	

Table 4 illustrates a binary logistic regression. By applying the regression analysis, it was aimed to find variables in a multivariate model explaining discrepancy. The category as well as duration of hospital stay were analysed by means of their influence on discrepancy. The regression shows that only the disease category significantly influences the discrepancy without the factor ‘hospital stay’. The category ‘diseases of the cardiovascular system ‘ serves as reference category. It can be assumed that solely the ‘category’ itself is relevant when supposing that the autopsy result differs from the clinical cause of death. The factors “disease category”, “age” and “new-born” were statistically significant. Diseases of the cardiovascular systemoccur in 60% of all discrepancies, but diseases of the urogenital tract were correctly diagnosed by 100%. Sex, BMI, and hospital duration had no significant impact on the occurrence of discrepancies.

**Table 4:** Binary logistic regression with discrepancy as a dependent variable.

	Odds Ratio	Lower CI	Upper CI	p- value
(Intercept)	0.785	0.626	0.984	0.036
Duration of Hospital stay in days (d)	1.001	0.992	1.010	0.871
Category				<0.0001
Tumour	0.245	0.145	0.398	<0.0001
Infectious disease	0.438	0.070	0.240	<0.0001
Respiratory	0.438	0.273	0.688	<0.0001
Digestive	0.484	0.288	0.795	0.005
Genitourinary	0.000		394.772	0.971
Other	0.343	0.077	1.122	0.105

**Fig 2:**
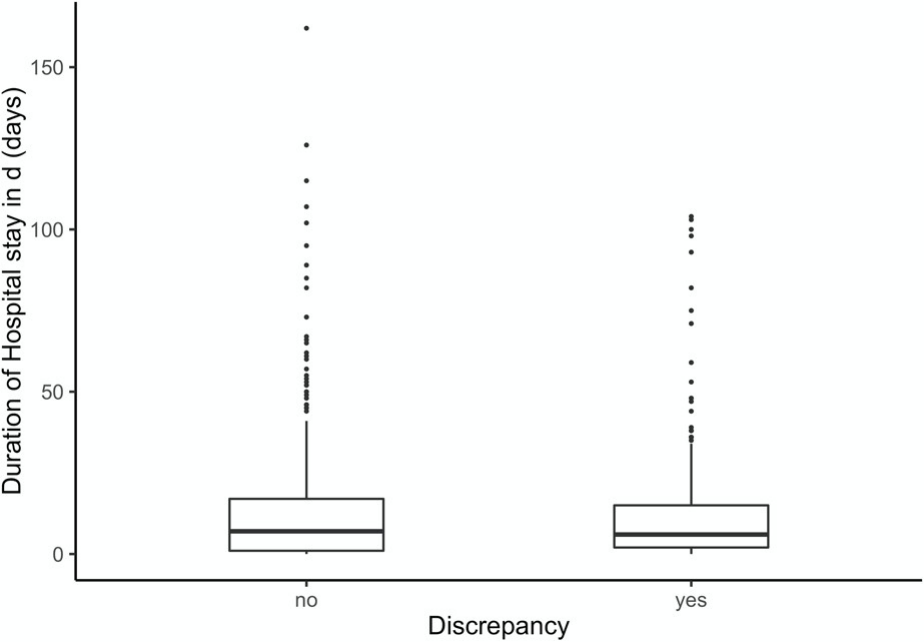
The Box-plot above represents the hospital duration compared to discrepancy groups.

In the second analysis in Table 2, new-borns were excluded. Patients with clinical cause of death as confirmed by autopsy stayed 9 days at hospital while patients with discrepancies between ante.- and post-mortem causes of death stayed 6 days or less in the hospital. Nonetheless, duration of hospital stay is an inhomogeneous variable, the longest hospital stay was 126 days (Fig 2). Table 3 shows that variables “duration of hospital stay” and “disease category” with and without discrepancy. The Kruskal Wallis test demonstrated significant results when comparing duration of hospital stay among disease categories.

We investigated within the respective disease category whether there was a significant difference in the duration of hospital stay with regard to correctly and incorrectly diagnosed cause of death, or not. There were no significant differences in the length of in-hospital stay between the groups “discrepancy” vs. “no discrepancy” when categorized to the disease groups. The regression analysis underlines these results (Table 4). Statistical significance was demonstrated in the disease category, but not for duration of hospital stay. In the univariate analyse the hospital duration is significant. Univariate logistic regression showed that the length of in-hospital stay was an independent predictor of discrepancies between clinically diagnosed causes of death and autopsy findings. Patients with diseases of the circulatory system stayed significantly shorter than patients with tumour (p = 0.0001), infections (p<0.0001), diseases of the respiratory system (p = 0.0001) or diseases of the digestive system (p = 0.005). Patients with diseases of the cardiovascular system represent the majority (41.9%), being the most frequently incorrectly diagnosed (62.5%).

Table 5 shows more detailed in which area the most frequent discrepancies occur. The three most common discrepant clinical diagnoses were “sepsis” (17.3%), “heart failure” (10.3%), and “unclear” (7.5%), while autopsy revealed “heart failure”, “sepsis” and “myocardial infarctions” were the three most frequent causes of death at autopsy. Clinical diagnoses and autopsy results overlap, and sepsis remains the most common clinical diagnosis, followed by heart failure. A further majority of cases are “unclear”. Sepsis was common in the clinical diagnoses, being the most serious form of complication of infection in several organs and organ systems. “Heart failure” was also common in both the clinical diagnosis (10.3%) and autopsy reports (23.6%).

**Table 5:** Most common clinical vs. most common autopsy diagnoses.

	Clinical diagnosis	ICD	N	%	Autopsy results	ICD	N	%
1.	Sepsis	A41.9	210	17,3	Heart failure	I50.9	286	23,6
2.	Heart failure	I50.9	125	10,3	Sepsis	A41.9	149	12,3
3.	Unclear	R99	91	7,5	Myocardial infarction	I21.9	95	7,8

## Discussion

The aim of our study was to identify discrepancies between clinical diagnoses and autopsy findings as well as explanations for the occurrence of these discrepancies. The rate of discrepancies between clinical diagnoses and postmortem results is still high, and autopsy rates in Germany are declining. from almost 60% in the 1960s to approximately 10% between 2000–2003 [9].

Distinct levels of causes of death include the underlying cause of death, immediate cause of death, antecedent cause of death and contributory causes of death. We used the immediate cause of death. i.—e. the final result of the autopsy, and compared this result with the leading clinical diagnosis immediately before death as documented in the autopsy request form. Clinico-pathological hospital autopsies—in contrast to medico-legal autopsies in unclear or non-natural deaths, which were not included in this study–are carried out in cases of natural deaths if ante-mortem consent of the deceased or postmortem consent of the relatives is obtained are performed for quality measurement purposes and medical as well as clinico-pathological education. Even though there is a downward trend (not only) for hospital autopsies, we are able to report significant discrepancies between ante-mortem clinical diagnosis and postmortem diagnosis. Autopsy is mandatory for retrospective quality assessment of clinical diagnosis [4,5,10,11], and also for training or teaching, or like in our case, for medical statistics [12–14]. High discrepancy rates between clinical diagnosis and hospital autopsies show that autopsies still play an important role in uncovering the underlying cause of death [15].

We identified variables such as age and disease categories, as being relevant for presenting discrepancies. Patients of the discrepancy group were three years older at median than patients without discrepancy.

Diseases of the digestive system were incorrectly diagnosed in only 25% of all cases. No significant differences in causes of death occurred with regard to sex, men and women were equally represented. Most deaths occurred from diseases of the—cardiovascular system. The older the patient was, the higher was the risk of discrepancy between clinical diagnosis and the underlying autopsy result, most probably due to higher incidence of diseases of the cardiovascular system system in older age, being misdiagnosed because of similar clinical symptoms, e. g. acute aortic dissection and myocardial infarction are the most frequently missed diagnoses [4,15,16]. We conclude that focused ante-mortem attention is needed with regard to acute chest pain, and these patients must be evaluated to distinguish for example between acute coronary syndrome, pulmonary embolism, acute aortic syndrome, esophageal rupture, or tension pneumothorax [17]. Autopsy is a relevant quality improvement tool, and the presented data have relevant clinical impact [12,13,18]. Several studies have outlined the importance of autopsy for clinical practice and. have shown a high rate of discrepancies between clinical and postmortem diagnoses [19–21]. Despite this fact, autopsy rates are declining. One reason for this is certainly the advances in medical imaging and additional clinical tests in everyday life [22].

The diagnostic and technical progress in medicine have changed the conditions which lead to diagnostic discrepancies ante- and postmortem [23]. Lack of time for appropriate diagnostic tests can have a high impact on misdiagnoses [18,24]. Buschmann et al. described cases of trauma-related death, where diagnostic assessment was limited due to short stay in the emergency room or required cardiopulmonary resuscitation [24]. In a systemic review by Shojania et al, it was reported that principal diagnoses and causes of death had been determined without autopsy [25]. According to Shojania et al. major errors are defined as clinically missed diagnoses involving a principal underlying disease or primary cause of death. Yet autopsy remains a valuable tool to evaluate the diagnostic and therapeutic process [25]. With regard to our results, we conclude that interdisciplinary data analysis should be combined with forensic and pathological autopsy results. Goldman et al. reported that even accurate diagnostic test can give misleading results, as to increase its sensitivity for detecting abnormalities, its specificity will decline by means of test interpretation. Moreover, clinicians might not accurately report due to misunderstanding of test results. Especially when case history is unavailable and external findings are not present, determining the cause of death is difficult [26,27]. Apart from differences in natural causes of death as described above, non-natural causes of deaths might also be missed [27]

A meta-analysis of 5,863 hospital autopsies showed a prevalence of misdiagnoses ranging from 5.5% to 100% [28]. Myocardial infarctions, pulmonary embolism and pneumonia were the leading diseases [28]. We observed 73.9% (n = 822) patients without discrepancies between autopsy and clinical diagnosis.

Autopsy stills remain the most important intervention to determine the cause of death [23].

## Limitation of the study

- Data were derived only from a single institution in a retrospective study design.
- The inter- and intra-observer bias might be relevant as pathologists may interpret the clinical findings in a different way.
- In cases of myocardial infarction macroscopic changes occur after 4–6 hours of survival. Even with histological examinations, short-time survival of myocardial infarctions can be missed at autopsy.

## Conclusion

Future strategies to reduce diagnostic errors in living patients should focus on older patients with cardiovascular diseases to improve patients” safety. Even in our era of high-tech diagnostic possibilities in the living, autopsies remain indispensable for medical quality control and proof for diagnostic accuracy–in individual cases as well as in epidemiological issues, despite small and further declining autopsy rates. Elevated public and health policy levels of consciousness for the overall value of post-mortem medical interventions in terms of inclining autopsy rates are desirable in the future.
